# Occupational stress, cardiovascular vulnerability and sudden cardiac death in police officers: mechanisms and the protective role of structured exercise

**DOI:** 10.3389/fpubh.2026.1838794

**Published:** 2026-05-14

**Authors:** Jing Xu, Junjun Zhang

**Affiliations:** Department of Police Tactics, Fujian Police College, Fuzhou, China

**Keywords:** autonomic imbalance, cardiovascular vulnerability, occupational health strategy, occupational stress, police officers, structured physical exercise, sudden cardiac death

## Abstract

Sudden cardiac death (SCD) is a leading global mortality cause, with police officers as a high-risk group due to unique chronic occupational stressors—chronic psychological hypervigilance, circadian disruption, sudden high-intensity physical exertion under stress, and delayed health-seeking behavior. This review elucidates the “dual-hit model” linking occupational stress to SCD: chronic stress-induced cardiovascular vulnerability (via autonomic imbalance, myocardial electrophysiological instability, endothelial dysfunction, systemic inflammation) plus acute operational triggers. We evaluate structured physical exercise as a targeted modulator reversing stress-related cardiovascular remodeling via autonomic rebalancing, anti-inflammation, electrophysiological stabilization, and metabolic/circadian regulation. An integrated multi-layered occupational health strategy is proposed: cardiovascular risk stratification, evidence-based exercise prescription, autonomic/recovery monitoring, psychological resilience training, and organizational workload optimization. Critical knowledge gaps and future directions (prospective cohort studies, biomarker validation, exercise dose–response exploration, wearable technology, randomized controlled trials) are identified. This review provides a conceptual framework for preventing SCD in police officers and high-stress populations, highlighting structured exercise as a core occupational cardiovascular risk management component.

## 1 Introduction

Sudden cardiac death (SCD) remains a leading cause of mortality worldwide, primarily driven by malignant ventricular arrhythmias in the context of structural or electrical heart disease. While classic cardiovascular risk factors (hypertension, dyslipidemia, diabetes) are well-established drivers of long-term cardiac disease progression, acute fatal cardiac events typically require additional physiological triggers. Research attention has thus increasingly shifted to occupational stress and autonomic dysregulation as proximal determinants of sudden cardiac events (1).

Police officers are a uniquely vulnerable occupational group, chronically exposed to psychological hypervigilance, traumatic incidents, unpredictable operational demands, sleep deprivation, circadian disruption, and episodic high-intensity physical exertion (2). These stressors form a sustained occupational stress environment that progressively disrupts cardiovascular homeostasis (3).

Epidemiological studies have consistently documented elevated rates of cardiovascular morbidity and mortality among police officers compared with the general population (4). However, the mechanistic pathways linking occupational stress to increased SCD risk have not yet been fully synthesized and elucidated (5).

Emerging evidence indicates that chronic occupational stress induces a state of cumulative physiological burden (elevated allostatic load), characterized by persistent sympathetic activation, hypothalamic–pituitary–adrenal (HPA) axis dysregulation, reduced heart rate variability (HRV), endothelial dysfunction, systemic inflammation, and electrophysiological instability (6). Over time, these abnormalities generate a vulnerable cardiovascular substrate, which—when combined with acute stressors (e.g., physical confrontation, emergency response)—may precipitate malignant ventricular arrhythmias or acute coronary events, ultimately leading to SCD (7).

Notably, structured physical exercise exerts multi-system cardiovascular protective effects, including autonomic rebalancing, anti-inflammatory modulation, improved endothelial function, enhanced cardiac reserve, and stabilization of myocardial electrophysiology (8). These adaptive changes suggest that regular structured exercise may counteract stress-induced cardiovascular vulnerability and reduce SCD risk in high-stress occupations (9).

This review aims to: (1) characterize the specific occupational stress profile of police work; (2) elucidate the mechanistic pathways linking chronic occupational stress exposure to SCD; and (3) evaluate the potential of structured physical exercise as a targeted protective intervention to mitigate stress-related cardiovascular risk. By integrating insights from occupational health science, cardiovascular pathophysiology, and exercise biology, we propose a conceptual framework for understanding and preventing SCD in high-stress professional populations (10).

## 2 Occupational stress profile of police officers

Police work is distinguished not merely by high workload, but by a distinct, sustained stress architecture comprising psychological, physiological, circadian, and situational stressors (11, 12). These stressors are temporally unpredictable, biologically repetitive, and cumulative, creating a persistent state of cardiovascular strain. Dissecting their core components and physiological impacts is critical to understanding elevated SCD risk in this population (13).

### 2.1 Chronic psychological hypervigilance and traumatic exposure

Police officers operate in environments that demand constant threat appraisal; even during routine patrols, cognitive vigilance must remain heightened (14). This persistent anticipatory alertness is mediated physiologically by sustained activation of the sympathetic nervous system (SNS) (15). In addition, officers are frequently exposed to traumatic incidents, including violence, severe injury, and sudden death (3). Repeated trauma exposure has been associated with a series of adverse physiological and psychological changes: elevated basal catecholamine levels, flattened diurnal cortisol rhythms, reduced heart rate variability (HRV), and a higher prevalence of post-traumatic stress symptoms (16).

Reduced HRV—particularly diminished high-frequency power, a marker of vagal withdrawal—has been consistently linked to increased arrhythmic vulnerability and cardiovascular mortality (17). Chronic autonomic imbalance therefore represents a central intermediate phenotype that bridges occupational stress and elevated SCD risk (18). Importantly, this hypervigilant state does not resolve immediately after duty hours; autonomic dysregulation often persists into rest periods, impairing the body’s ability to achieve physiological recovery (19).

### 2.2 Shift work, circadian disruption, and sleep deprivation

Law enforcement work frequently involves rotating shifts, overnight duty, and unplanned emergency call-outs, all of which lead to circadian misalignment and sleep restriction (20). These disruptions exert profound and multifaceted effects on the cardiovascular system (21). Short sleep duration (<6 h per night) and irregular sleep schedules are associated with: elevated nocturnal blood pressure, impaired endothelial-dependent vasodilation, increased circulating inflammatory markers (e.g., IL-6, CRP), insulin resistance, and reduced HRV (22).

Circadian disruption also directly alters myocardial electrophysiology: experimental sleep deprivation studies have demonstrated QT interval prolongation and an increased frequency of ventricular ectopy (21, 23–25). Moreover, nocturnal sympathetic surges that occur during insufficient sleep may lower the threshold for malignant ventricular arrhythmias (26). From a mechanistic perspective, chronic sleep disruption contributes to the accumulation of allostatic load and may accelerate the development of a vulnerable myocardial substrate, further increasing SCD risk (20).

### 2.3 Episodic high-intensity physical exertion under stress

Unlike athletes who perform high-intensity exercise under controlled, structured conditions, police officers may be required to transition abruptly from a sedentary state to maximal physical exertion during pursuits, suspect restraint, or emergency interventions—all while under acute psychological stress (27). This sudden, unplanned physical exertion in a stress state triggers a cascade of acute hemodynamic and neurohormonal changes: rapid surges in heart rate and blood pressure, a sharp increase in myocardial oxygen demand, transient coronary shear stress, and elevated circulating catecholamines (28).

In individuals with subclinical coronary atherosclerosis, these acute hemodynamic spikes may precipitate coronary plaque rupture or vasospasm (29). Simultaneously, catecholamine excess enhances triggered electrical activity within the ventricular myocardium, potentially initiating ventricular tachyarrhythmias (30). This pattern aligns with the well-established clinical concept that SCD often occurs during or shortly after intense physical or emotional stress, particularly in individuals with an underlying pre-existing cardiovascular vulnerability (31).

### 2.4 Organizational culture and delayed health-seeking behavior

Police culture in many regions places a strong emphasis on endurance, resilience, and emotional stoicism (32). While these traits are operationally valuable for law enforcement work, they may unintentionally discourage officers from early reporting of cardiovascular symptoms such as chest discomfort, palpitations, presyncope, or excessive fatigue (33). Delayed medical evaluation and intervention can allow underlying structural or electrical cardiac abnormalities to remain undetected and progress, increasing long-term SCD risk (34, 35).

Additionally, the social stigma surrounding psychological stress and mental health conditions may prevent officers from seeking timely management of post-traumatic stress disorder (PTSD) and other stress-related psychological issues, which in turn perpetuates chronic autonomic imbalance. This sociocultural factor interacts synergistically with biological stress pathways, extending the duration of physiological strain and compounding cardiovascular risk in police officers (36–38).

## 3 From chronic occupational stress to cardiovascular vulnerability

SCD rarely occurs in a structurally and electrophysiologically normal heart (39). Instead, it typically reflects the interaction between an underlying vulnerable cardiovascular substrate and an acute triggering event (40). In high-stress occupations such as policing, chronic occupational stress may progressively generate this vulnerable substrate through converging neuroendocrine, inflammatory, vascular, and electrophysiological pathways (41). This pathological process can be conceptualized as stress-induced cardiovascular remodeling—a series of adaptive yet ultimately adverse changes to the heart and vascular system driven by persistent occupational stress (31, 42).

### 3.1 Persistent sympathetic overactivation and autonomic imbalance

Chronic psychological stress and hypervigilance trigger sustained activation of both the sympathetic–adrenal–medullary axis and the hypothalamic–pituitary–adrenal (HPA) axis, leading to long-term autonomic dysregulation with far-reaching cardiovascular consequences: elevated resting catecholamine levels, increased resting heart rate, reduced HRV (particularly diminished vagal tone), and blunted baroreflex sensitivity (43, 44).

Reduced HRV is an independent predictor of increased cardiovascular mortality and SCD risk (45). Mechanistically, diminished parasympathetic modulation lowers the ventricular fibrillation threshold and increases susceptibility to reentrant arrhythmias—two key precursors of malignant ventricular arrhythmias (46, 47). Chronic catecholamine excess also exerts direct adverse effects on myocardial tissue, promoting cardiomyocyte apoptosis, interstitial fibrosis, *β*-adrenergic receptor desensitization, and intracellular calcium handling abnormalities (48). These structural and cellular changes contribute to myocardial electrical heterogeneity, a critical prerequisite for the development of malignant ventricular arrhythmias.

### 3.2 Stress-induced myocardial remodeling and electrophysiological instability

Prolonged autonomic imbalance alters myocardial electrophysiology at multiple cellular and tissue levels, driving the development of an electrically unstable myocardium (49). This process manifests in three key ways:

#### 3.2.1 Ion channel modulation

Chronic stress exposure has been associated with altered potassium channel expression in cardiac myocytes, prolonged myocardial action potential duration, QT interval prolongation on the electrocardiogram, and increased dispersion of myocardial repolarization. Increased QT dispersion reflects spatial heterogeneity in myocardial repolarization times, a key factor that facilitates the formation of reentry circuits— the primary mechanism underlying most malignant ventricular arrhythmias (50–53).

#### 3.2.2 Triggered activity

Elevated circulating catecholamines enhance intracellular calcium cycling in cardiac myocytes. Abnormal calcium overload within the myocyte can generate delayed afterdepolarizations (DADs) and early afterdepolarizations (EADs)—abnormal electrical depolarizations that can act as ectopic pacemakers. In a susceptible myocardium, these triggered electrical activities may initiate sustained ventricular tachycardia or ventricular fibrillation, leading to SCD (54–57).

#### 3.2.3 Fibrotic substrate formation

Chronic inflammatory signaling and neurohormonal activation (driven by persistent stress) promote myocardial interstitial fibrosis. Fibrotic tissue disrupts normal myocardial electrical conduction pathways, increasing conduction delay and anisotropy. Both changes favor the formation and maintenance of reentrant arrhythmias, further stabilizing the electrically vulnerable myocardial substrate (58, 59). Collectively, these changes mean that chronic occupational stress may progressively remodel myocardial tissue into an electrically unstable state, priming the heart for malignant arrhythmias.

### 3.3 Endothelial dysfunction and atherosclerotic instability

Beyond its direct effects on myocardial electrophysiology, chronic occupational stress exerts profound adverse consequences on the vascular system, particularly the coronary arteries, driving endothelial dysfunction and atherosclerotic instability.

#### 3.3.1 Endothelial dysfunction

Sustained sympathetic activation reduces nitric oxide bioavailability in the vascular endothelium and increases oxidative stress—two key drivers of endothelial dysfunction. This impairs endothelium-dependent vasodilation and promotes pathological vasoconstriction, altering coronary blood flow regulation and increasing myocardial ischemia risk (60–62).

#### 3.3.2 Inflammatory activation

Elevated circulating inflammatory mediators (e.g., IL-6, TNF-α, CRP) have been consistently documented in individuals with chronic stress exposure. Systemic low-grade inflammation accelerates the development of atherosclerotic plaques in the coronary arteries and promotes plaque destabilization by weakening the fibrous cap and increasing lipid core size (63–66).

#### 3.3.3 Plaque vulnerability

Acute hemodynamic surges during stress episodes increase coronary shear stress. In patients with unstable atherosclerotic plaques—characterized by a thin fibrous cap, large lipid core, and increased inflammatory cell infiltration—this acute shear stress may precipitate plaque rupture and subsequent thrombus formation. Acute coronary occlusion due to plaque rupture and thrombosis may then trigger malignant ventricular arrhythmias, accounting for a substantial proportion of SCD cases in adult populations (67–70).

### 3.4 Circadian disruption and electrical vulnerability

Sleep deprivation and circadian misalignment, key components of police occupational stress, further compound cardiovascular vulnerability by exacerbating myocardial electrical instability and vascular dysfunction (71). Disrupted sleep patterns are associated with: elevated nocturnal sympathetic tone, impaired myocardial repolarization stability, an increased frequency of ventricular ectopy, and blunted nocturnal blood pressure dipping (non-dipping patterns) (72).

Circadian rhythms also play a critical role in regulating cardiac ion channel expression and autonomic balance; persistent circadian misalignment disrupts these regulatory processes, increasing arrhythmic susceptibility during the early morning hours—a well-documented peak time for SCD and acute coronary events in the general population. For police officers, this circadian-driven vulnerability is amplified by chronic sleep deprivation and overnight duty, further increasing their overall SCD risk (73, 74).

### 3.5 Acute trigger superimposed on a vulnerable substrate

The culmination of these chronic stress-induced pathological processes is a state of profound cardiovascular vulnerability, characterized by: reduced vagal tone, myocardial electrical heterogeneity, fibrotic myocardial remodeling, coronary plaque instability, and impaired endothelial function (75). When a police officer encounters an acute high-stress operational event—such as physical confrontation, emergency response, or high-speed pursuit—a rapid surge in catecholamines, blood pressure, and myocardial oxygen demand occurs.

In the presence of a pre-existing vulnerable cardiovascular substrate, this acute stress trigger may initiate a cascade of adverse events that lead to SCD, including: (1) induction of coronary plaque rupture and acute thrombosis; (2) precipitation of coronary vasospasm and myocardial ischemia; (3) initiation of triggered ventricular electrical activity; and (4) promotion of sustained reentrant ventricular tachyarrhythmia.

The interaction between chronic stress-induced cardiovascular vulnerability and acute operational stress triggers thus forms the core mechanistic basis of stress-associated SCD (76). This dual-hit model—vulnerable substrate plus acute trigger—provides a biologically coherent framework for understanding the elevated SCD risk observed in law enforcement personnel and other high-stress occupational populations (35).

## 4 Physical exercise as a biological modulator of stress-induced cardiovascular vulnerability

Although acute unplanned intense physical exertion may transiently increase cardiovascular risk in susceptible individuals, a large body of evidence demonstrates that structured, regular physical exercise induces adaptive cardiovascular remodeling that reduces overall SCD risk (77, 78). It is therefore essential to draw a clear distinction between two distinct forms of physical exertion in high-stress occupations: (1) unprepared, sudden maximal exertion under psychological stress—a high-risk scenario that can trigger SCD in vulnerable individuals; and (2) progressive, structured aerobic and resistance training under controlled conditions—a protective intervention that induces adaptive physiological changes.

In high-stress occupations such as policing, the absence of structured physical conditioning may amplify cardiovascular vulnerability during acute exertional events (79). Conversely, systematic exercise training may reprogram the body’s stress-response system and stabilize cardiovascular physiology, counteracting the adverse effects of chronic occupational stress (80). Below is a detailed analysis of the multiple protective mechanisms through which structured physical exercise modulates stress-induced cardiovascular vulnerability.

### 4.1 Autonomic rebalancing and increased vagal tone

One of the most robust and well-documented adaptive changes to regular aerobic training is enhanced parasympathetic (vagal) activity, which reverses stress-induced autonomic imbalance (81, 82). The cardiovascular benefits of this adaptation include: reduced resting heart rate, increased HRV (particularly the high-frequency components that reflect vagal tone), improved baroreflex sensitivity, and a reduction in sympathetic dominance at rest and during mild stress (83).

Enhanced vagal tone directly increases the ventricular fibrillation threshold and stabilizes myocardial repolarization, reducing the risk of malignant ventricular arrhythmias (84). Higher HRV is consistently associated with reduced cardiovascular mortality in both the general population and high-risk groups. In chronically stressed police officers, structured exercise therefore represents a powerful intervention to counteract stress-induced autonomic imbalance and restore sympathovagal equilibrium.

### 4.2 Anti-inflammatory and endothelial protective effects

Regular moderate-intensity exercise exerts systemic anti-inflammatory effects that counteract the chronic low-grade inflammation driven by occupational stress (85). These effects are mediated by: reduced baseline circulating levels of pro-inflammatory cytokines (IL-6, CRP), increased production of anti-inflammatory cytokines, improved mitochondrial efficiency (which reduces oxidative stress), and a direct reduction in systemic oxidative stress (86, 87).

Exercise also exerts direct protective effects on the vascular endothelium by enhancing endothelial nitric oxide synthase (eNOS) activity, which increases nitric oxide bioavailability and improves vascular compliance (88, 89). Improved endothelial function reduces the risk of coronary vasospasm, slows the progression of atherosclerotic plaque formation, and stabilizes existing atherosclerotic plaques (90)—thereby decreasing the likelihood of acute ischemic triggers of SCD, such as plaque rupture and thrombosis.

### 4.3 Myocardial structural and electrophysiological stabilization

Chronic structured exercise induces adaptive cardiac remodeling—often referred to as the “athlete’s heart”—which is fundamentally distinct from pathological hypertrophy driven by hypertension or heart failure (91, 92). This physiological remodeling is characterized by: increased stroke volume, improved diastolic function, enhanced myocardial perfusion, and improved intracellular calcium handling efficiency in cardiac myocytes (91).

Notably, regular physical conditioning has been associated with a series of electrophysiological benefits that reduce arrhythmic risk: a shorter QT interval, reduced QT dispersion, and a lower incidence of ventricular ectopy at rest and during mild exertion. Improved calcium cycling stability in cardiac myocytes reduces susceptibility to delayed afterdepolarizations (DADs) and early afterdepolarizations (EADs), thereby decreasing the arrhythmogenic potential of the myocardium. Collectively, these structural and electrophysiological changes stabilize the heart and reverse stress-induced myocardial electrical vulnerability (93–95).

### 4.4 Exercise as allostatic load reduction

Chronic occupational stress increases allostatic load—a cumulative physiological burden resulting from repeated activation of the body’s stress-response systems (96, 97). Structured physical training is a powerful intervention to reduce allostatic load in high-stress populations, through multiple mechanisms: improved stress resilience (the ability to cope with acute stress without excessive neurohormonal activation), faster autonomic recovery after acute stress events, reduced resting cortisol levels and a restoration of normal diurnal cortisol rhythms, and enhanced psychological coping capacity for occupational stressors (97–99).

Thus, structured exercise functions not merely as a form of cardiovascular conditioning, but as a systemic stress-modulating intervention that addresses the root cause of stress-induced cardiovascular vulnerability: the cumulative physiological burden of chronic occupational stress.

### 4.5 Proposed integrative model

The mechanistic link between chronic occupational stress, cardiovascular vulnerability, and SCD, as well as the protective role of structured physical exercise, can be conceptualized as a sequential pathway:

Chronic Occupational Stress → Autonomic Imbalance + Systemic Inflammation + Endothelial Dysfunction + Myocardial Electrical Instability → Cardiovascular Vulnerability Substrate → Acute Stress Trigger → Sudden Cardiac Death.

Structured physical exercise intervenes at multiple upstream nodes in this pathway, exerting a multi-level protective effect:

- Restores autonomic balance and increases vagal tone.
- Reduces systemic pro-inflammatory signaling and oxidative stress.
- Stabilizes myocardial electrophysiology and reverses stress-induced electrical heterogeneity.
- Improves endothelial integrity and reduces atherosclerotic instability
- Enhances physiological and psychological stress recovery, reducing allostatic load.

By modifying and reversing the stress-induced vulnerable cardiovascular substrate, structured exercise reduces the probability that an acute operational stress trigger will culminate in a malignant ventricular arrhythmia or acute coronary event, thereby lowering overall SCD risk. This multi-level modulation suggests that structured exercise programs should be considered a central component of occupational cardiovascular risk management in high-stress professions such as law enforcement.

## 5 From mechanistic insight to occupational health strategy

The mechanistic framework presented above clarifies that SCD risk in police officers arises from the interaction between cumulative stress-induced cardiovascular vulnerability and acute operational triggers (35). Therefore, effective preventive strategies must aim not only to detect established cardiac disease, but also to modify and reverse the upstream stress-induced cardiovascular vulnerability substrate (42). Translating these mechanistic insights into clinical and occupational practice requires an integrated approach that combines cardiovascular screening, structured exercise programming, physiological stress monitoring, and recovery optimization—all tailored to the unique occupational stress profile of law enforcement personnel (100).

### 5.1 Cardiovascular risk stratification and baseline assessment

Before implementing exercise-based interventions, a comprehensive cardiovascular risk evaluation is essential to stratify risk and avoid exercise-induced adverse events (101, 102). Recommended components of a baseline assessment for police officers include:

- Resting 12-lead electrocardiography (ECG).
- Serial blood pressure measurements (e.g., averaged from two readings on each of three separate visits over several weeks, as recommended by standard hypertension guidelines) and a comprehensive metabolic profile (fasting glucose, lipid panel, liver and renal function).
- Systematic assessment of traditional cardiovascular risk factors (smoking, alcohol use, obesity, family history).
- Detailed evaluation of family history of SCD and heritable cardiac conditions (e.g., long QT syndrome, hypertrophic cardiomyopathy).
- Exercise tolerance testing for individuals identified as high-risk (e.g., older officers, those with multiple traditional risk factors).
- Echocardiographic screening when clinically indicated (e.g., abnormal resting ECG, positive exercise tolerance test, symptoms of cardiac disease).

For officers over a defined age threshold (e.g., 40 years) or with multiple cardiovascular risk factors, more advanced evaluation may be considered, including ambulatory ECG monitoring (Holter monitoring) to detect silent ventricular ectopy or arrhythmias, and coronary computed tomography angiography (CCTA) to screen for subclinical coronary atherosclerosis.

This risk stratification allows for the classification of officers into three distinct groups, each with a tailored intervention approach:

- Low-risk individuals: suitable for unsupervised moderate-intensity conditioning programs;
- Intermediate-risk individuals: requiring supervised exercise programs with regular monitoring;
- High-risk individuals: needing targeted medical management of underlying cardiac conditions before participation in any structured exercise program.

This personalized risk stratification minimizes the paradoxical risk of exercise-triggered cardiac events in susceptible individuals while ensuring that all officers receive a safe and effective exercise intervention.

### 5.2 Structured exercise prescription principles

Based on current evidence from cardiovascular medicine and exercise physiology, the following evidence-based principles should guide the design of structured exercise programs for law enforcement personnel, balancing protective cardiovascular adaptation with operational relevance and safety:

#### 5.2.1 Frequency

A minimum of 150 min per week of moderate-intensity aerobic activity, distributed across 3–5 sessions to avoid excessive fatigue and ensure adequate recovery (101, 103). Shorter, more frequent sessions are preferred for officers with irregular shift schedules, as they improve adherence and reduce the risk of missed workouts.

#### 5.2.2 Intensity

Moderate intensity (approximately 50–70% of maximal aerobic capacity, or a rating of perceived exertion of 5–7/10) is sufficient to induce the key autonomic, endothelial, and anti-inflammatory benefits of exercise while minimizing the acute risk of arrhythmias or myocardial ischemia (104, 105). High-intensity interval training (HIIT) may be considered for low-risk officers but should be avoided in intermediate or high-risk groups due to the acute hemodynamic stress it imposes (106, 107).

#### 5.2.3 Progressive overload

A gradual escalation of exercise intensity, duration, or volume over time—typically 5–10% every 2–3 weeks—prevents abrupt sympathetic surges and allows the cardiovascular system to adapt slowly to increased physical demands (108). This progressive approach is critical for reducing the risk of overtraining and exercise-induced injury.

#### 5.2.4 Combined modality

Incorporation of both aerobic training (e.g., running, cycling, swimming) and resistance training (e.g., bodyweight exercises, weight lifting) enhances the metabolic, vascular, and musculoskeletal benefits of exercise. Resistance training should focus on functional movements relevant to law enforcement work (e.g., strength, endurance, agility) to improve operational performance while reducing cardiovascular risk (109–111).

#### 5.2.5 Recovery emphasis

Adequate post-exercise recovery time should be integrated into all programs, particularly for officers with irregular shifts or overnight duty. This includes active recovery (e.g., light walking, stretching) after intense sessions, and at least one full rest day per week to allow for physiological repair and adaptation (112, 113).

Importantly, conditioning programs should simulate the functional operational tasks of police work (e.g., pursuit, restraint, carrying heavy equipment) under controlled and progressive conditions. This functional fitness training reduces the physiological shock of sudden unplanned physical exertion during real-life operational events, further bridging the gap between exercise adaptation and occupational safety.

### 5.3 Monitoring autonomic and recovery status

Given the central role of autonomic imbalance in stress-related SCD, continuous physiological monitoring of autonomic function and recovery status may offer additional value in optimizing exercise programs and identifying early signs of excessive cumulative stress (114, 115). Potential non-invasive monitoring tools for law enforcement personnel include:

- Continuous HRV tracking (via wearable devices) to assess autonomic balance and stress recovery
- Longitudinal monitoring of resting heart rate trends (an elevated resting heart rate is a marker of excessive stress and poor recovery)
- Objective assessment of sleep duration and quality (via wearable devices or sleep diaries)
- Wearable-based stress indices (combining HRV, skin conductance, and activity data) to quantify real-time occupational stress load

While these tools are not diagnostic and should not replace clinical evaluation, they can help identify states of cumulative fatigue or insufficient physiological recovery in individual officers, allowing for timely adjustments to exercise volume, intensity, or work workload. This real-time monitoring ensures that exercise programs remain adaptive and safe, even in the face of the unpredictable operational demands of police work.

### 5.4 Stress management and psychological resilience integration

Structured physical exercise alone cannot fully mitigate the adverse effects of chronic occupational stress; exercise interventions must therefore be integrated with targeted stress management and psychological resilience training to address both the biological and psychological components of stress-induced cardiovascular vulnerability. Integrated programs should incorporate the following components:

- Cognitive stress-management training: mindfulness-based stress reduction (MBSR), cognitive behavioral therapy (CBT), and breathing exercises to reduce acute stress reactivity and improve cognitive coping skills.
- Trauma-informed psychological support: specialized mental health care for officers with PTSD/trauma-related symptoms (individual counseling, group therapy) to address the psychological root of chronic autonomic imbalance.
- Peer-support systems: structured programs to reduce mental health/cardiovascular symptom stigma, encouraging early reporting and help-seeking behavior.
- Sleep hygiene education: tailored training for shift-working officers to improve daytime sleep quality, reduce circadian misalignment, and avoid sleep-disrupting behaviors (excessive caffeine, pre-bed screen time).

Enhancing psychological resilience and stress management skills may indirectly stabilize autonomic regulation and reduce chronic sympathetic activation, amplifying the cardiovascular protective effects of structured exercise and creating a synergistic intervention for stress-induced SCD risk reduction (114, 116, 117).

### 5.5 Organizational-level considerations

Occupational cardiovascular risk reduction is unlikely to succeed if limited to individual-level interventions (e.g., exercise, stress management); meaningful and sustainable risk reduction requires organizational-level changes that address the structural drivers of occupational stress in law enforcement. Police organizations should consider the following evidence-based organizational strategies to reduce stress-induced cardiovascular risk:

- Minimize consecutive night shifts and limit the number of overnight shifts per month to reduce circadian misalignment and sleep deprivation.
- Whenever operationally feasible, a recovery interval of at least 12 h should be considered, particularly for high-risk individuals following a documented high-intensity event (e.g., physical pursuit, restraint, emergency response). For low- or intermediate-risk officers, shorter recovery periods may be acceptable, but clinical judgment and autonomic monitoring (e.g., HRV) should guide return to duty. 0020Facilitate on-duty physical training time by incorporating structured exercise into patrol and duty schedules, removing barriers to adherence such as time constraints and work demands.
- Implement organizational policies that encourage early reporting of cardiovascular and psychological symptoms, with no negative career consequences for seeking medical or mental health care.

Most importantly, police organizations must undertake a cultural reframing of health-seeking behavior: from a sign of weakness to a core component of operational readiness (118, 119). A healthy, physically fit, stress-managed officer is a more effective, resilient law enforcement professional; framing occupational health protection as a critical operational priority is essential to improving preventive intervention adherence and reducing workforce-wide SCD risk (18, 120).

### 5.6 Implementation model: a multi-layered prevention strategy

Based on the mechanistic insights and evidence-based principles outlined above, an integrated, multi-layered prevention model for reducing SCD risk in police officers is proposed. This model targets both the biological stress-induced cardiovascular vulnerability substrate and the occupational stress triggers, with five interconnected layers that build on each other to create a comprehensive and sustainable intervention:

Layer 1—baseline cardiovascular screening: comprehensive risk stratification and baseline assessment to identify underlying cardiac disease and classify risk;
Layer 2—structured exercise conditioning: evidence-based, personalized exercise programs tailored to risk stratification and operational functional fitness needs;
Layer 3—continuous recovery monitoring: non-invasive wearable monitoring of autonomic function, sleep, and stress load to optimize exercise and work workload;
Layer 4—psychological stress support: integrated stress management, trauma-informed care, and peer support to reduce psychological stress and improve resilience;
Layer 5—organizational workload optimization: structural and cultural organizational changes to reduce the structural drivers of occupational stress and improve adherence to preventive interventions.

**Figure 1:**
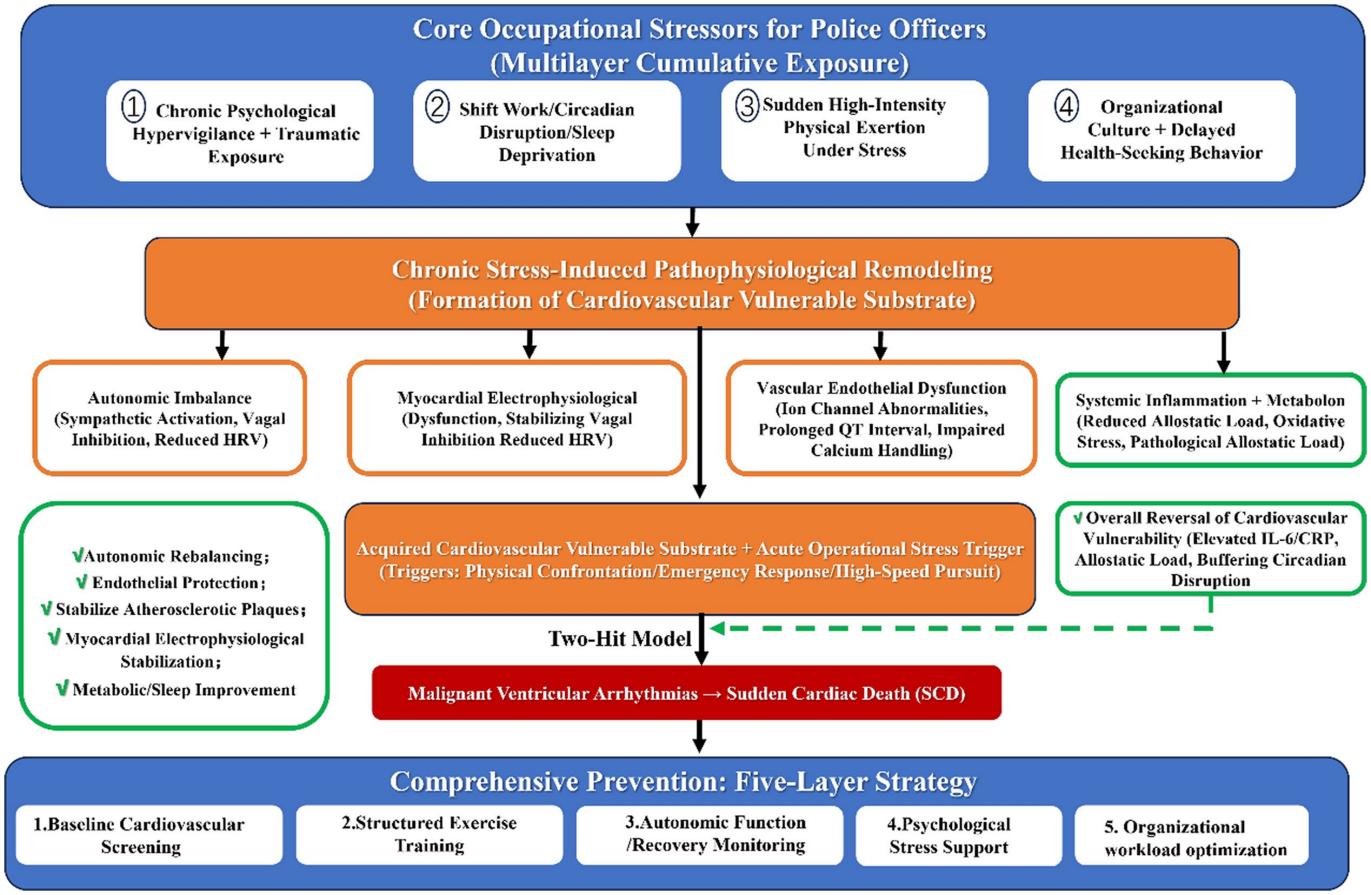
Conceptual model of stress-induced cardiovascular vulnerability and exercise-mediated protection in police officers.

By simultaneously targeting biological vulnerability and occupational stress exposure patterns, this multi-layered model addresses both the substrate and trigger components of SCD risk in a synergistic manner. It is a flexible model that can be adapted to the unique needs of different police departments (e.g., urban vs. rural, large vs. small) and ensures that preventive interventions are operational relevant, sustainable, and aligned with the core mission of law enforcement (Figure 1).

Chronic occupational stress exposure (psychological hypervigilance, traumatic events, shift work, sleep deprivation, sudden high-intensity exertion) leads to persistent sympathetic activation, reduced vagal tone, HPA axis dysregulation, and increased allostatic load. These physiological alterations promote electrophysiological instability, endothelial dysfunction, inflammatory activation, and myocardial remodeling, collectively forming a vulnerable cardiovascular substrate. Acute operational triggers may precipitate malignant ventricular arrhythmias or acute coronary events in this vulnerable state, resulting in sudden cardiac death. Structured physical exercise modulates upstream autonomic imbalance, reduces inflammation, improves endothelial function, and stabilizes myocardial electrophysiology, thereby attenuating substrate vulnerability and lowering event probability.

## 6 Conclusion

Sudden cardiac death in high-stress occupations such as law enforcement is not an unpredictable, isolated event, but the culmination of progressive cardiovascular vulnerability shaped by police work’s unique, sustained occupational stress architecture. Four key stressors—chronic psychological hypervigilance, circadian disruption/sleep deprivation, episodic high-intensity physical exertion under stress, and delayed health-seeking behavior—converge to generate a cascade of adverse physiological changes: autonomic imbalance, endothelial dysfunction, systemic inflammatory activation, myocardial remodeling, and electrophysiological instability. Over time, these changes form a stress-induced vulnerable cardiovascular substrate that primes the heart and vascular system for malignant arrhythmias and acute coronary events.

When an acute operational trigger (intense physical confrontation, emergency response, high-speed pursuit) supervenes on this vulnerable state, the probability of malignant ventricular arrhythmia or acute coronary event increases substantially. This dual-component model (chronic stress-induced cardiovascular vulnerability + acute operational stress triggers) provides a biologically coherent explanation for the elevated SCD risk observed in police officers compared with the general population.

Notably, structured physical exercise emerges not merely as a general health recommendation, but as a targeted biological modulator capable of attenuating and reversing stress-induced cardiovascular remodeling. Through its multi-system protective effects—autonomic rebalancing, anti-inflammatory modulation, endothelial protection, myocardial electrophysiological stabilization, and sleep/circadian regulation—regular structured conditioning reduces the stress-induced vulnerable cardiovascular substrate upon which acute triggers act, lowering overall SCD risk.

Effective cardiovascular risk reduction in police officers therefore requires a multidimensional, integrated strategy combining personalized cardiovascular risk stratification, evidence-based structured exercise programming, continuous physiological recovery monitoring, psychological resilience training, and organizational workload optimization. This approach addresses both substrate and trigger components of SCD risk synergistically, ensuring interventions are clinically effective, operationally relevant, and sustainable for law enforcement personnel.

Ultimately, reframing occupational health protection as a core element of operational readiness and law enforcement effectiveness is the single most important step for achieving sustainable workforce safety in high-stress professions. Investing in police officers’ cardiovascular and psychological health not only reduces SCD risk, but also enhances workforce resilience, performance, and well-being—creating a win-win for officers and the communities they serve. Ultimately, reframing occupational health protection as a core element of operational readiness and law enforcement effectiveness may be the single most important step for achieving sustainable workforce safety in high-stress professions. By investing in the cardiovascular and psychological health of police officers, law enforcement organizations not only reduce SCD risk but also enhance the resilience, performance, and well-being of their workforce—creating a win-win for both officers and the communities they serve.
